# The Microbiome of Temporal Arteries

**DOI:** 10.20411/pai.v4i1.270

**Published:** 2019-02-12

**Authors:** Gary S. Hoffman, Ted M. Getz, Roshan Padmanabhan, Alexandra Villa-Forte, Alison H. Clifford, Pauline Funchain, Madhav Sankunny, Julian D. Perry, Alexander Blandford, Gregory Kosmorsky, Lisa Lystad, Leonard H. Calabrese, Charis Eng

**Affiliations:** 1 Center for Vasculitis Care and Research; Department of Rheumatic and Immunologic Diseases; Cleveland Clinic; Cleveland, Ohio; 2 Genomic Medicine Institute; Lerner Research Institute; Cleveland Clinic; Cleveland, Ohio; 3 Taussig Cancer Institute; Cleveland Clinic; Cleveland, Ohio; 4 Cole Eye Institute; Cleveland Clinic; Cleveland, Ohio; 5 Division of Rheumatology, University of Alberta, Canada; 6 Department of Genetics and Genome Sciences; Case Western Reserve University School of Medicine; Cleveland, Ohio; 7 Germline High Risk Focus Group; CASE Comprehensive Cancer Center; Case Western Reserve University School of Medicine; Cleveland, Ohio

**Keywords:** vasculitis, giant cell arteritis, microbiome

## Abstract

**Objective:**

A role for microorganisms in giant cell arteritis (GCA) has long been suspected. We describe the microbiomes of temporal arteries from patients with GCA and controls.

**Methods:**

Temporal artery biopsies from patients suspected to have GCA were collected under aseptic conditions and snap-frozen. Fluorescence *in situ* hybridization (FISH) and long-read 16S rRNA-gene sequencing was used to examine microbiomes of temporal arteries. Taxonomic classification of bacterial sequences was performed to the genus level and relative abundances were calculated. Microbiome differential abundances were analyzed by principal coordinate analysis (PCoA) with comparative Unifrac distances and predicted functional profiling using PICRUSt.

**Results:**

Forty-seven patients, including 9 with biopsy-positive GCA, 15 with biopsy-negative GCA and 23 controls without GCA, were enrolled. FISH for bacterial DNA revealed signal in the arterial media. Beta, but not alpha, diversity differed between GCA and control temporal arteries (*P* = 0.042). Importantly, there were no significant differences between biopsy-positive and biopsy-negative GCA (*P > 0.99*). The largest differential abundances seen between GCA and non-GCA temporal arteries included Proteobacteria (P),* Bifidobacterium* (g), *Parasutterella* (g), and *Granulicatella* (g) [Log 2-fold change ≥ 4].

**Conclusion:**

Temporal arteries are not sterile, but rather are inhabited by a community of bacteria. We have demonstrated that there are microbiomic differences between GCA and non-GCA temporal arteries, but not between biopsy-positive and biopsy-negative GCA.

## INTRODUCTION

Giant cell arteritis (GCA) is the most common large vessel vasculitis in adults, with an estimated incidence of 15-25 cases/100,000 among persons > 50 years old [1]. The most frequently affected vessels are the extracranial branches of the carotid arteries, resulting in headache, scalp tenderness, jaw claudication, and visual aberration or blindness. Temporal artery biopsies represent the most readily accessible source of tissue for confirmation of diagnosis; however, they may fail to reveal inflammatory infiltrates in up to 50% of cases, due to the presence of skip lesions or the predominance of large vessel disease [2]. Post-mortem and imaging studies have also revealed that involvement of the aorta and its primary branch vessels is common, if not universal, in GCA [3-5]. Indeed, in a post-mortem study of the aorta and primary branch vessels in 13 consecutive patients with GCA, all had features of vasculitis in spite of treatment with corticosteroids in 9 of 13 patients for several months to up to 9 years duration [3]. Although patients with GCA usually respond quickly to treatment with high dose glucocorticoids, relapses occur frequently when doses are tapered, suggesting that the underlying driver of inflammation has not been addressed [6, 7].

Cellular infiltrates in GCA include activated vascular dendritic cells, which attract Th1 and Th17 lymphocytes and activated macrophages to the arterial wall. The antigen(s) responsible for initial activation of dendritic cells has not yet been identified [1]. Interferon gamma is the hallmark cytokine of the Th1 response and is a cytokine triggered in response to intracellular pathogens [8]. It is therefore plausible that infectious agents may be providing antigenic stimulation in GCA.

A role for infection in GCA is supported by the observation of cyclical peaks in disease incidence [9]. Multiple viral [10-13] and bacterial [14-16] agents have previously been implicated; however, attempts at pathogen detection by epidemiologic, focused genetic, and immunohistochemical approaches have failed to provide consistent results. Recent reports of varicella zoster virus in temporal artery biopsies in GCA are intriguing, but require additional confirmation [17-20].

Sequencing of the bacterial-specific 16S ribosomal RNA gene from human tissue is a sensitive and culture-independent method for both pathogen and commensal detection, allowing for a more comprehensive and unbiased description of the temporal artery microbiome in GCA.

## MATERIALS AND METHODS

### Sample Accrual and Collection

We prospectively enrolled consecutive consenting patients undergoing temporal artery biopsy for evaluation of possible GCA, under a study protocol conducted in compliance with the Helsinki Declaration and approved by the Institutional Review Board at the Cleveland Clinic. All participants provided written informed consent. The authors GSH and CE together have full access to the data and take responsibility for its integrity and data analysis. The temporal artery biopsies were collected under strictly aseptic conditions by a team of ophthalmologists. Biopsies did not include skin. Biopsies were split, with one-half sent for routine histopathological review and one-half snap-frozen under sterile technique for microbiome analysis. Patients were classified according to clinical phenotype, including corticosteroid use, clinical symptoms, co-morbidities and histopathology results as either biopsy-positive GCA (histopathology confirming inflammatory infiltrates and compatible clinical presentation), biopsy-negative GCA (histopathology without inflammatory infiltrates, but meeting the American College of Rheumatology 1990 Classification criteria for GCA [21] and a persistent clinical diagnosis of GCA at least 3 months post-biopsy), or as controls (patients in whom the diagnosis of GCA and other forms of vasculitis were subsequently ruled out). Following >3 years of collection, all samples were processed at the same time. Laboratory-based microbiome investigators were kept unaware of the patients' clinical and pathological diagnoses.

### Fluorescence In Situ Hybridization (FISH)

Two samples (TA6 Control and TA13 GCA) were examined by FISH to identify the possible presence of bacteria within the vessel wall. Tissue had been snap-frozen for 16S rRNA gene analysis (described below) and also formalin-fixed and paraffin-embedded per routine protocols. FISH for bacterial 16S rRNA was performed using a previously published protocol [22]. Sections of 2 µm were cut and mounted on coated microscope slides. Sections were deparaffinized and then subjected to cell wall and protein degradation with incubations in lysozyme followed by proteinase K. Sections were hybridized, for 3.5 hours, with 100µM EUB 338, a previously published oligonucleotide complementary to a universally conserved region of the bacterial 16S rRNA gene. Following this step, sections were counterstained with 0.025% (weight per volume) concanavalin A-Alexa Fluor 594 (Integrated DNA Technologies, Coralville, IA) for 20 minutes. Finally, sections were mounted with Vectashield-DAPI (Vector Laboratories, Burlingame, CA) and dried overnight. Concanavalin A is used as a counterstain for glycoproteins, while DAPI is used for identification of human nuclei. Images were acquired using a Leica TCS-SP2 spectral laser scanning confocal microscope operated with Leica Confocal Software (Leica Microsystems, GmbH, Wetzlar, Germany).

Fluorescence intensities from signals of FISH-positive bacteria were measured using Image J (NIH). The mean intensity was calculated by subtracting the background intensity for the green channel from the measured intensity within the region of interest (within the media, ie, not external to the adventitia).

### 16S Ribosomal RNA-Gene Sequencing of Temporal Artery Biopsies

Total deoxyribonucleic acid (DNA) was isolated using the AllPrep DNA/RNA Isolation Kit according to the manufacturer's protocol (Qiagen, Valencia, CA) with minor modifications [23]. Briefly, all beads, tubes, and non-enzymatic reagents were treated with UV light for 30 minutes prior to use; samples were digested with 20 µL of 20 ng/µL Proteinase K (Roche Diagnostics Corp., Indianapolis, IN) at 65°C for 1 hour, then transferred to 0.1 mm glass beaded tubes. After this the samples were homogenized using the TissueLyser LT (Qiagen). The quality and purity of the isolated total DNA were confirmed spectrophotometrically using a NanoDrop 2000 device (Fisher Scientific SAS, Illkirch, France). DNA concentration was quantified using the Qubit 2.0 instrument applying the Qubit dsDNA HS Assay (Life Technologies). Extracted DNA samples were stored at -20°C.

Bacterial 16S rRNA-gene amplification and library construction were performed according to the 16S Metagenomic Sequencing Library Preparation guide from Illumina (Forest City, CA). In brief, 2 µL total DNA was amplified using primers targeting the 16S V3 and V4 region (Illumina) at 95°C for 5 minutes, followed by 35 cycles at 95°C for 30 seconds, 56°C for 30 seconds, and 72°C for 30 seconds with a final extension at 72°C for 10 minutes. The 16S rDNA amplicons were run out on a 1% agarose gel, size-selected at 450-500 bp, and gel-purified using QIAquick Gel Purification kit (Qiagen). A second round of PCR was performed to add Nextera XT indices (Illumina) to purified amplicons. Indexed PCR products were cleaned with Ampure XP beads (Beckman Coulter, Inc., Brea, CA) and resulting libraries quantified with the QuantiFluor dsDNA system according to the manufacturer's protocol (Promega, Madison, WI). Samples were then normalized to 10nM and pooled into sequencing libraries. Pooled V3-V4 amplicon libraries were sequenced using the Illumina MiSeq platform with V3 reagent kit. The 300-bp paired end reads for each sample were demultiplexed and quality checked using FastQC 0.11.3.

Temporal artery specimens were stored in sterile containers. Sterilized water was aliquoted into the containers and then removed and 16S rRNA gene sequencing performed, revealing no contamination.

### Microbiome Analysis

A hybrid post-sequencing analysis methodology using QIIME and MICCA was adopted, and preprocessing was performed in QIIME and open-reference operational taxonomic unit (OTU) picking was performed with MICCA and Phyloseq [24, 25]. After the biom files were created, downstream analysis was performed with QIIME. The 250-bp Illumina paired-end reads were merged with FLASH [26], and low-quality reads (Phred < 20) were filtered out using the split_libraries.py command in QIIME (version 1.9) [27]. MICCA vsearch (version 1.9.5) [28] was utilized for clustering the sequences with a threshold of 97% similarity, and representative sequences were classified using RDP classifier (version 2.11) [29]. Multiple sequence alignments were performed using MUSCLE (version 3.8.31) [30, 31] against the Greengenes database (version 13.8) [32], filtered at 97% similarity, and FastTree (version 2.1.8) was used for phylogenetic tree construction [33].

Data clean-up was done by removing the singletons and discarding taxa represented in fewer than 5% of total samples. The rarefaction value was set to 8,467 reads per sample to reduce sampling heterogeneity, and computation of alpha (Shannon diversity index) and beta diversity measures (unweighted UniFrac distances) were performed with PhyloSeq in R. Alpha diversity measures species richness (number of taxa) within a single microbial ecosystem. Beta diversity can be represented by UniFrac distances which describe similarities and dissimilarities between bacterial communities using phylogenetic information, taking into account the number of taxa and relative abundances within each taxon. F-tests based on sequential sums of squares derived from 1,000 permutations on UniFrac distance matrices were performed with the null hypothesis that there is no difference in community structure between groups. Note that PCoA and the calculation of *P* values are measurements of clustering strength. This is not based on linear correlations (because this is not linear). Differences (and the *P* value) are derived from measuring differences between UniFrac distances. To find which taxa are most likely to explain the differences between our clinical groupings, taxa summaries and differential abundances were analyzed with DESeq2. This algorithm estimates variance-mean dependence in count data and tests for differential expression based on a model using the negative binomial distribution. Differentially abundant taxa that were statistically significant using an alpha of 0.05 and exceeded a Log2-fold change of ±2 were visually represented on box plots. A heatmap was generated from the top differentially dominant OTUs using the Bray-Curtis distance methods and where the plot was created using pheatmap in R.

We analyzed functional composition of microbiomes using the PICRUSt 1.0.0-dev bioinformatics package [34]. We filtered out all de novo OTUs and used this OTU table as our input into the PICRUSt algorithm, which calculates contributions of various OTUs to known biological pathways based on evolutionary modeling. The OTU picking was performed against the Greengenes (gg_13_8) database using a 97% similarity threshold. Welch's *t* test was used to calculate *P* values, and corresponding Storey q-values were used to control for the false discovery rates associated with multiple testing. These values were calculated using DESeq2 and LEfSe and visualized as a cladogram or bar plot using Phyloseq or LEfSe [35].

## RESULTS

### Patients

Forty-seven patients were enrolled in the study, including 9 patients with a final diagnosis of biopsy-positive GCA, 15 patients with biopsy-negative but clinically confirmed GCA, and 23 additional patients in whom the diagnosis of GCA was ruled out by histology and clinical course (Table 1). Median age at the time of biopsy was 71 years for those with GCA and 73 for non-GCA controls (*P* = 0.5). Two-thirds of the patients were female (*P* = 0.065 between GCA and non-GCA patients) [Table 1]. Thirty-eight of 49 patients (78%) had been receiving daily prednisone (mean dose > 50mg/day) prior to temporal artery biopsies (89% in the GCA biopsy-positive group, 86% in the GCA biopsy-negative group, and in 71% of controls). There was no difference between groups in prednisone use (*P* > 0.35), dose (*P* > 0.5) or duration of treatment (*P* = 0.56) [Table 1].

**Table 1.** Patient baseline demographics at the time of biopsy

	Controls (23)	Bx-GCA* (15)	Bx+ GCA (9)	Total (47)
Age (years)	72.9+/-9.4	71.2+/-2.4	75.5+/-3.2	71.9+/-8.9
Female, no. (%)	15 (65%)	9 (60%)	4 (44%)	28 (60%)
Race, no. White (%)	21 (91%)	12 (80%)	8 (89%)	41 (87%)
Vascular Symptoms, no. (%)	16 (70%)	12 (80%)	8 (89%)	36 (77%)
Systemic Symptoms, no. (%)	2 (9%)	6 (40%)	1 (11%)	9 (19%)
ESR (mm/hr) (mean+/-SD, range)	39+/-6	48+/-7	32+/-9	40+/-25
CRP (mg/dl) (mean+/-SD, range)	1.8+/-1.1	4.5+/-1.5	5.6+/-1.6	3.5+/-4.5
Prednisone use (%)	15 (65%)	12 (80%)	8 (89%)	35 (74%)
Prednisone use >50mg/d (mean prior to biopsy)	12 (52%)	6 (40%)	5 (56%)	23 (49%)
Duration of prednisone (days) [mean+/-SD]	23+/-35	14+/-6	32+/-50	22+/-35
Other immunosuppression	2 (9%)**	0 (0%)	0 (0%)	2 (4%)

* Biopsy-negative, but clinically confirmed positive GCA.

** One patient each was receiving low dose methotrexate for rheumatoid arthritis and another for granulomatosis with polyangiitis.

### Histopathology

Temporal artery biopsies of the 9 patients with a final diagnosis of biopsy-proven GCA revealed arteritis, with mononuclear cell inflammatory infiltrates localized to the adventitia and media, fragmentation of the internal elastic lamina, and varying degrees of intimal proliferation and fibrinoid necrosis. All biopsies from patients who were biopsy-negative with clinically positive GCA and 20 of 23 TA from controls without GCA revealed arteriosclerosis, with intimal thickening and rare, focal calcification; 3 TA from controls without GCA were normal.

### Bacterial DNA Detection in Temporal Arteries by FISH

**Figure 1.**
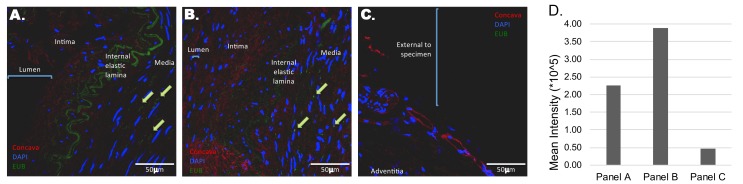
**Distribution of bacterial DNA in temporal arteries.** Tissue sections were probed with fluorescently labeled oligonucleotide probes against bacterial DNA (green). Sections were counterstained with DAPI (blue) and Concanavalin A (red) to delineate nuclei and glycoproteins, respectively. Sections were scanned by confocal microscopy. In a control temporal artery (A), bacterial DNA is scattered throughout the media, with select examples highlighted by green arrows. Notably, no/negligible bacterial DNA staining is apparent in the lumen or intima (A, bar graph). The green channel emitted from the internal elastic lamina is a result of autofluorescence. In a temporal artery with histopathological evidence of GCA (B), bacterial DNA is scattered throughout the media and at a higher mean intensity than control (bar graph). Arterial layers are more disorganized and less distinct compared to a control temporal artery, as evidenced by weak autofluorescence and less distinct internal elastic lamina. There is an absence of bacterial DNA at the external edge of a GCA-involved temporal artery specimen (C, bar graph).

Sterile, fresh frozen temporal artery biopsies from a control patient (TA6C) and a patient with GCA (TA13G) were fixed and paraffin-embedded. FISH using an oligonucleotide probe specific for bacterial 16S rRNA revealed the presence of multiple single bacteria in the media of both control and GCA-involved arteries (Figure 1). No FISH signal corresponding to the presence of bacteria was apparent in the intimal layer, arterial lumen, or external border of the specimen. Corroborating the FISH microscopy, the mean intensity for the FISH-positive bacteria (Figure 1, bar graph) was higher in GCA-involved arteries compared to that of the control temporal artery (Figure 1B vs 1A), and no signal was ascertained at the external edge of a GCA-involved temporal artery (Figure 1C).

### Microbiome in Temporal Arteries from Patients with and without Giant Cell Arteritis

**Figure 2.**
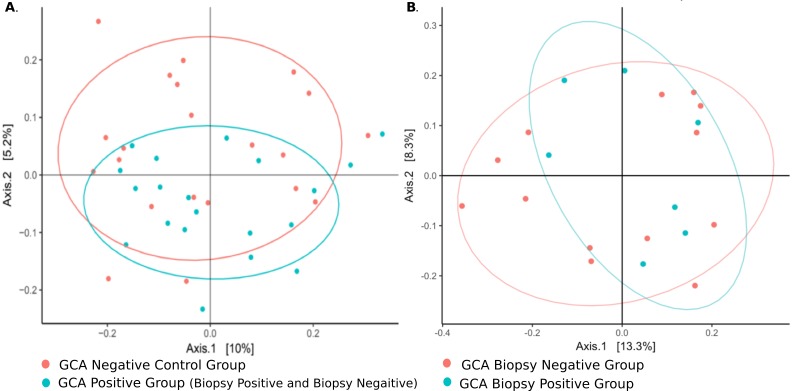
**Microbiomes from TAs with biopsy-positive and biopsy-negative GCA cluster together but differently from those from control patients.** Principal component analysis (PCoA) of TA microbiomes. (A) β-diversity (not α, data not shown), differs between GCA and control groups (*P* = 0.042). (B) There were no statistically significant differences between TA microbiomes in those with biopsy-positive GCA vs those with biopsy-negative/clinically positive GCA (*P* > 0.99).

Culture-independent, long-read genomic sequencing was used to characterize the entire microbial communities of temporal arteries from the 47 research participants. After sequencing and quality control, 4 samples were excluded from further analyses because of low read counts, leaving 43 samples comprising 20 with GCA (7 biopsy-positive, 13 biopsy-negative) and 23 without GCA. There were no alpha diversity differences between GCA and non-GCA temporal artery microbiota. In contrast, beta-diversity, as measured by unweighted UniFrac distances, differed between the GCA and non-GCA groups (*P* = 0.042, Figure 2A). Of note, there were no statistically significant differences between temporal arteries from those with biopsy-positive GCA and those with biopsy-negative GCA (*P* = 1.0, Figure 2B).

**Figure 3.**
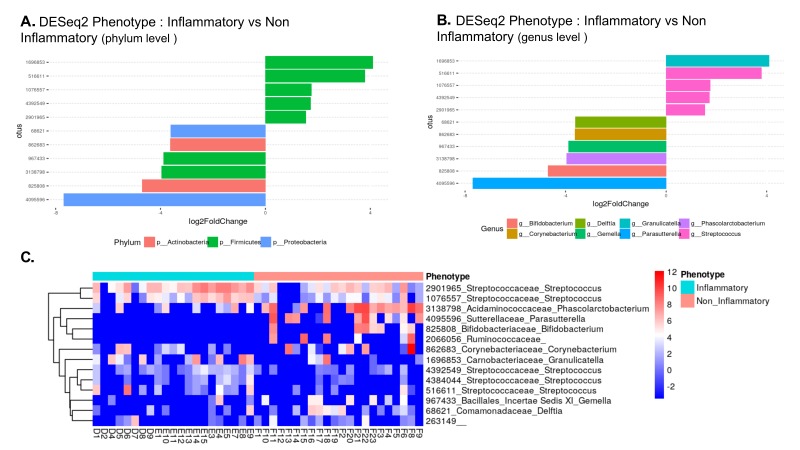
**Most differentially abundant taxa in temporal artery biopsies from patients with GCA and from control patients.** (A) Bar blot representation from DESeq2 showing the most over-represented (+) and under-represented (-) phyla in TAs from patients with GCA compared to TAs from controls. (B) Bar blot representation from DESeq2 showing the most over-represented (+) and under-represented (-) genera in TAs from patients with GCA compared to TAs from controls. (C) Heat map of bacterial communities in TA with GCA (“inflammatory” blue bar) compared to those without GCA (“noninflammatory” pink bar) based on the top dominant OTUs. Columns and rows represent samples and dominant OTUs, respectively. Row names on the right of the heat map include Green Genes ID followed by family and genus.

In order to gain insight into the microbial inhabitants of temporal arteries, we compared the relative abundances of bacterial OTUs within temporal artery biopsies with and without GCA (Figure 3). At the phylum level, there were at least 2 classes of Firmicutes relatively over-represented (> + 4) in GCA temporal arteries compared to those without GCA, although there were 2 other classes of Firmicutes relatively under-represented in temporal arteries with GCA versus temporal arteries without GCA (< -4, Figure 3A). Proteobacteria and Actinobacteria were relatively under-represented in temporal artery samples from patients with GCA compared to those without GCA (< -4; Figure 3A). At the genus level, *Granulicatella* and *Streptococcus*, both belonging to phylum Firmicutes, were relatively over-represented whereas *Parasutteralla*, belonging to phylum Proteobacteria, and *Bifidobacterium*, belonging to phylum Actinobacteria, were relatively under-represented in temporal artery samples from patients with GCA compared to those without GCA (Figure 3B).

### Predicting Functional Consequences of Differing Microbial Composition in GCA- and Non-GCA-Associated Temporal Arteries

**Figure 4.**
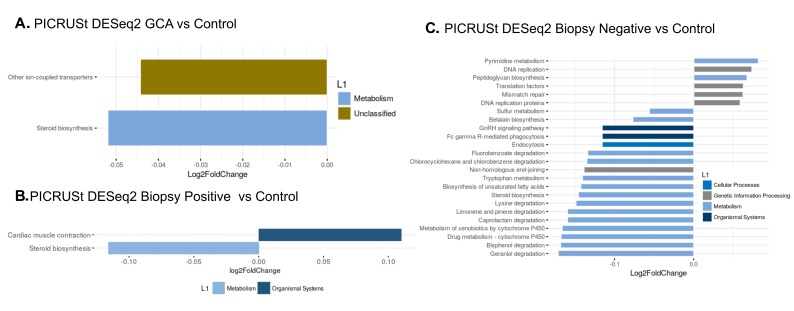
**Predicted functional pathways differentially represented in GCA TA compared to control (non-GCA) TA.** Representation of PICRUSt DESeq2 analysis yielding relatively under-represented functional pathways in (A) GCA TA compared to control TA, (B) biopsy-positive GCA TA compared to control TA and (C) biopsy-negative GCA TA versus control TA.

We analyzed microbiomes by predicting their functional contributions to their host environments using the PICRUSt algorithm. There was relative downregulation of ion-coupled transporters and steroid biosynthesis pathways in the group with GCA (combining biopsy-positive and biopsy-negative) compared to temporal arteries from non-GCA controls (all *P* < 0.05, Figure 4A). When only the biopsy-positive GCA temporal arteries were compared to non-GCA controls, the steroid biosynthesis pathway was relatively downregulated while the cardiac muscle contraction pathway was relatively upregulated (Figure 4B). While the steroid biosynthesis pathway remained relatively downregulated in biopsy-negative GCA compared to controls, there were multiple other pathways that were relatively upregulated and downregulated (Figure 4C). Interestingly, predicted downregulation of metabolism pathways appears to be found between both biopsy-positive GCA compared to control and biopsy-negative GCA compared to control.

## DISCUSSION

Our most reliable and important observation is that temporal arteries are not sterile, as previously assumed, but rather are inhabited by communities of bacteria in both the control and diseased state. Interestingly, the microbiomes of biopsy-negative, clinically confirmed GCA temporal arteries were similar to those of biopsy-positive GCA temporal arteries, suggesting a potential underlying similarity of temporal arteries from patients with GCA not reflected in histopathologic review. Together, microbial communities in biopsy-positive and biopsy-negative GCA temporal arteries were also distinct from those in control GCA-negative samples. This observation raises an important question regarding why there are histopathologic differences between biopsy-positive and biopsy-negative individuals with GCA since the microbiome between these subsets is similar. The answer is unknown. One might speculate that if microbiomes played a role in pathogenesis of GCA, that role may be permissive, and at a later uncertain time interval, be followed by a histologically apparent inflammatory response. If such were the case, “skip lesions” in GCA biopsies, which are well known, may result from a GCA step-wise inflammatory response. Our study did not address this possibility.

We believe that our GCA microbiome study is unique because of collecting and maintaining tissues using aseptic techniques, avoiding formalin fixation, paraffin embedding, and contamination with known skin and external environmental organisms and not disclosing patient diagnoses to our lab-based colleagues. Environmental contamination had been a concern of Bhatt and colleagues, who had identified *Propionibacterium acnes* and *Escherichia coli as* the most abundant microorganisms found in both formalin-fixed paraffin-embedded temporal arteries from patients with GCA and in controls [36]. Both quantitative and qualitative microbiome data were similar in their GCA cases and controls. Our study also differs in regard to clear qualitative differences noted between GCA and control samples. The differences in microbiome between GCA and non-GCA temporal artery specimens also supports the conclusion that contamination was not likely to have affected these specimens which were processed in identical fashion and analyzed at the same time. This is corroborated by our FISH studies which demonstrate bacterial nucleic acid within the arterial walls, without any bacteria external to the adventitia nor in the arterial lumen.

We were concerned about the effects of treatment on our analyses. It is generally recommended that once the diagnosis of GCA is considered, treatment with corticosteroids should not be delayed while awaiting temporal artery biopsy [5]. Consequently, it was expected that most of our patients (74%) had been treated prior to biopsy. However, in comparing corticosteroid treated and untreated cases, treatment did not appear to influence the microbiome.

Bhatt *et al* also note that indications for temporal artery biopsies in control patients are another factor that could impact GCA studies [36]. All biopsied individuals usually have had symptoms or findings suggestive of GCA, leading their physician to arrange for this procedure. Thus, investigators should be concerned about whether controls are truly GCA biopsy-negative but clinically positive. We tried to minimize this risk by following patients for at least 3 months post-biopsy to ensure that while untreated, features of GCA did not emerge and initial symptoms had resolved or could be attributed to alternative diagnoses. We therefore have a high degree of confidence that our controls did not have undiagnosed GCA.

Our results suggest that there does not appear to be a single bacterial pathogen characteristic of GCA. Species previously implicated in GCA pathogenesis such as *Mycoplasma pneumonia* [14],* Chlamydia pneumonia* [16], and *Burkholderia pseudomallei-like* organisms [15] were not found in our dataset. Given the high person-to-person species variability in our study, the inconsistency of named bacterial species in prior publications is not surprising. Additionally, many prior studies used formalin-fixed tissue, which is known to cause DNA cross-linking, nucleic acid shearing, reduction in yield, and sequencing artifacts [37]. We believe our fresh-frozen temporal artery samples, collected aseptically and specifically for microbiomic studies, most closely represent the actual bacterial constituents in temporal arteries.

Our findings included differences in microbiome content and density for the phylum Firmicutes, which was relatively over-represented, and Proteobacteria and Actinobacteria, which were relatively under-represented in temporal arteries from patients with GCA compared to controls. While differences were found in functional pathways between GCA and non-GCA cases, the significance of those changes is uncertain.

The notion of a vascular microbiome is neither new nor novel [38]. Many studies have demonstrated the presence of both bacteria and viruses within the walls of large and medium-sized blood vessels. Most compelling are the findings of bacteria in the lipid-rich core of plaques and within smooth muscle cells of atherosclerotic aortas and coronary arteries. Recent reports have also raised questions about the potential role of microbes in non-atherosclerotic arteries and apparently normal vessels. The most compelling of these studies are those that are metagenome-DNA-based. One study included 56 fresh, sterile aortic aneurysm samples from patients with atherosclerosis and non-atherosclerotic disease [39, 40]. Using PCR with universal 16S rRNA primers, bacterial DNA was isolated from about 90% of samples. Ten samples were selected for speciation from patients with Marfan's syndrome, idiopathic aortitis, aortic coarctation, and mechanical/degenerative aneurysms. All but 1 specimen revealed the presence of multiple bacterial species [39]. While metagenomic techniques are unbiased and more sensitive, they cannot determine whether the detected DNA is from living organisms or are remnants of microbes or contaminants. These unresolved questions can be applied to our findings in temporal artery biopsies as well and represent universal limitations of such studies.

Our study does have important strengths. It is the first to be performed on surgically sterile temporal arteries that were maintained under strict aseptic conditions throughout processing. Microbiome studies were performed by some of the authors without knowledge of clinical or pathological diagnoses. Arguing against contamination are first, the FISH studies that revealed the absence of bacterial DNA on the adventitial or luminal surfaces of specimens, and second, differences in microbial communities in patients with GCA versus controls (beta diversity). In addition, specimens from clinically diagnosed but biopsy-negative patients with GCA were not eligible for the study until at least 3 months of follow-up during which other diagnoses did not become apparent and satisfactory responses to corticosteroid therapy were well documented. Conversely, controls were not classified as such until similar follow-up revealed alternative diagnoses.

Our observations here are most important in emphasizing that temporal arteries are not sterile and that quantitative and qualitative microbiome and metabolic differences exist between those vessels in patients with GCA and controls. Localization of bacterial DNA using FISH indicates that bacteria are present within the temporal artery itself, and not within luminal deposits or due to external contamination at the surface of the sample. In fact, the external edges of excised temporal arteries displayed a remarkable absence of bacterial DNA. This finding is supported by another also suggesting the existence of a variety of bacteria in temporal arteries [17]. However, despite these suggestive observations, we have not proven that these findings play a role in the pathogenesis of GCA or are a result of substrate modifications due to GCA-mediated vessel injury.
